# A rare case of hepatoid adenocarcinoma of the lung

**DOI:** 10.1111/crj.13724

**Published:** 2023-12-10

**Authors:** Xuejian Sun, Jialin Liu, Ting Hu, Yefan Wu, Hao Zhang

**Affiliations:** ^1^ The First Clinical Medical College of Lanzhou University Lanzhou China; ^2^ Department of Radiology the First Hospital of Lanzhou University Lanzhou China

**Keywords:** HAL, hepatoid adenocarcinoma, hepatoid adenocarcinoma of the lung, rare lung cancer

## Abstract

Hepatoid adenocarcinoma of the lung is a special type of primary origin in the lung with obvious pathological features and short survival time. However, standard treatment guidelines have not yet been established. Herein, we report a case of hepatoid adenocarcinoma with the primary lesion located in the left upper lung. The tumour size was reduced after four cycles of combined therapy. Subsequent postoperative pathology confirmed complete remission.

## Graphical abstract

We report a case of hepatoid adenocarcinoma with the primary lesion located in the left upper lung. The patient was treated by four cycles of combined therapy, and the tumour had significantly reduced in size. Subsequent postoperative pathology proved that the patient had achieved a complete remission.

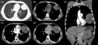

## 1 INTRODUCTION

Hepatoid adenocarcinoma of the lung (HAL) is a rare type of primary origin in the lung with obvious pathological features of hepatocellular carcinoma.^1^ In 1990, HAL was first reported by Ishikura et al^2^ in five patients with α‐fetoprotein expression and was considered similar to hepatocellular carcinoma and produce AFP. Nagai et al^3^ believed that gastric hepatoid adenocarcinoma can be diagnosed by the histomorphology of hepatoid adenocarcinoma, because it does not depend on the production of AFP. Although most cases of hepatoid adenocarcinoma are associated with AFP levels, an elevated AFP is only suggestive.^4^, ^5^

The clinical symptoms of HAL are non‐specific, and the treatment options are unclear. The current treatment methods depend on the primary site.^6^ Herein, we report the case of a patient with pulmonary hepatoid adenocarcinoma who achieved complete remission after receiving neoadjuvant chemotherapy, immunotherapy and targeted treatment. Thus, the present study aimed to enrich the available treatment information on HAL.

## 2 CASE REPORT

**FIGURE 1:**
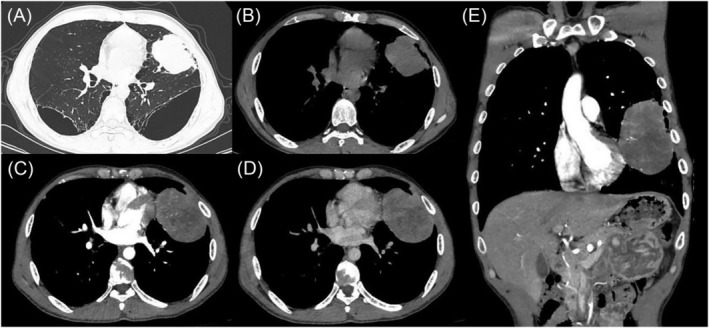
(A,B) Non‐contrast chest computed tomography (CT) scan revealed a soft tissue in the left upper lobe, measuring 47 × 39 mm in size. (C–E) Three months later, contrast‐enhanced CT showing the mass appeared inhomogeneous enhancement, which was larger than before, measuring 91 × 61 mm in size.

A 46‐year‐old man presented to our hospital with complaints of intermittent chest pain on the left side for 3 months, accompanied by a non‐irritating cough and sticky white phlegm. Specifically, it was a stabbing pain behind the sternum, occurring two to three times a day, lasting for several seconds each time and worsened after deep breathing but resolved on their own. The patient had a smoking history of 45 pack‐years of and 20 years of alcohol consumption but without any remarkable relevant family medical history. Non‐contrast computed tomography (CT) images revealed a lobulated mass in the inferior lingual segment of the left upper lobe of the lung; the longest cross‐sectional area measured 47 × 39 mm (Figure 1A,B). However, the patient declined puncture examination. Three months later, his symptoms exacerbated. Contrast‐enhanced CT images revealed an inhomogeneous enhancement of the mass, with bronchial truncation and identification of vascular structures within. The mass was larger, and the maximum cross‐sectional area became 91 × 61 mm (Figure 1C–E). No metastasis was found in the brain MR or bone nuclide scan. Abdominal CT scans revealed intrahepatic calcification, renal cyst and kidney stones.

**FIGURE 2:**
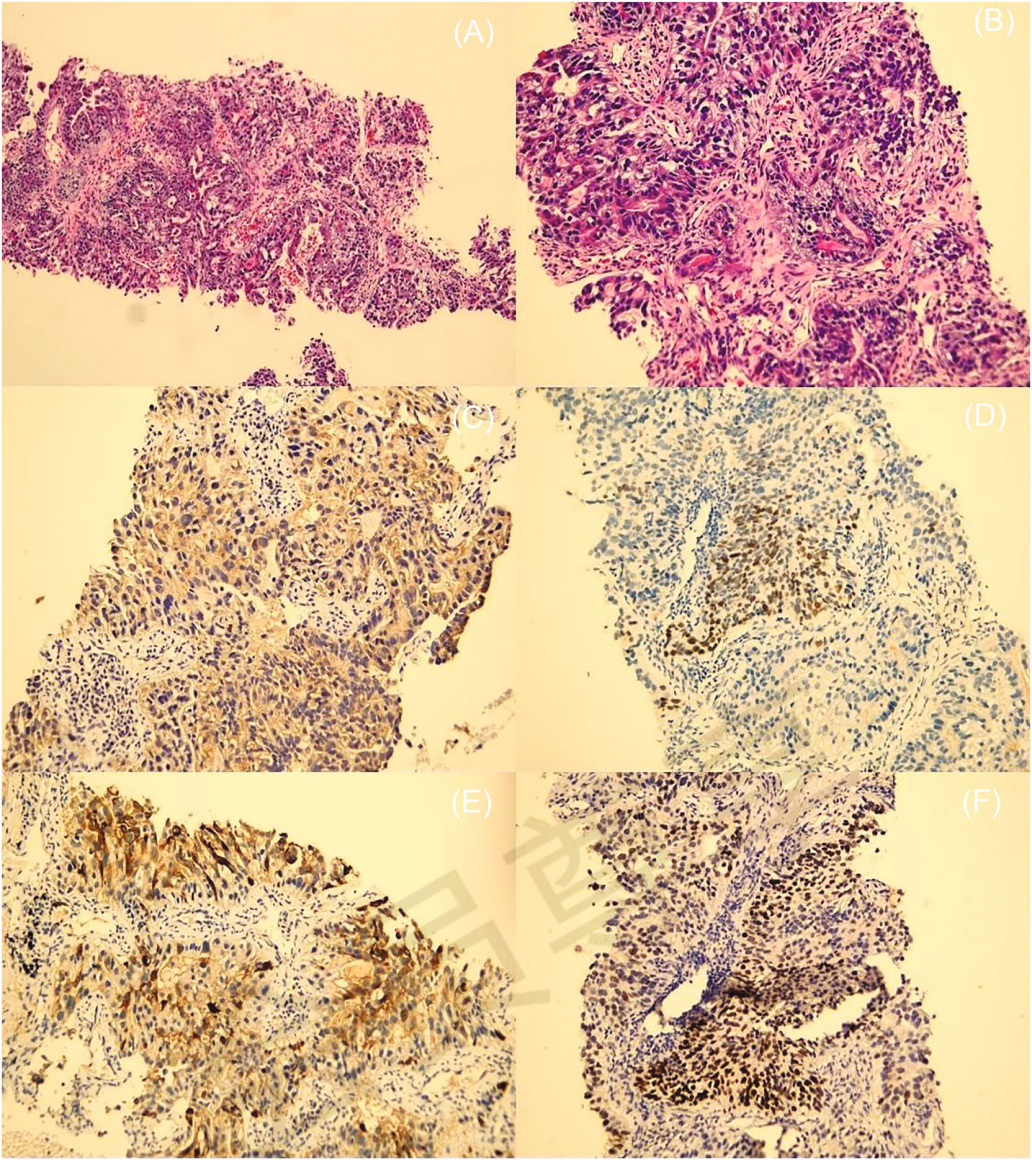
Histological features. (A,B) Haematoxylin and eosin (HE) indicated that the tumour tissue was arranged in irregular glandular tubular, sieve and nest‐like arrangement. The nuclear atypia was obvious, and the cytoplasm in some areas was clear, showing infiltrative growth. Hepatocyte (+) (C), SALL4 (+) (D), Syn (+) (E), TTF‐1(+) (F).

Serum carcinoma embryonic antigen (CEA), cytokeratin‐19‐fragment (CYFRA21‐1), carbohydrate antigen 242(CA242) and neuron‐specific enolase (NSE) were elevated, and AFP was 687 U/mL. The patient underwent percutaneous lung biopsy under CT guidance and routine paraffin sectioning. For haematoxylin–eosin staining, the tumour tissue was arranged in irregular glandular tubular, sieve and nest‐like arrangement. The nuclear atypia was obvious, and the cytoplasm in some areas was clear, showing infiltrative growth (Figure 2A,B). Immunohistochemistry staining demonstrated hepatocyte (+), glypican‐3 (+), cytokeratin 7 (CK7) (+), spalt‐like transcription factor 4 (SALL4) (+), synaptophysin (Syn) (+), caudal‐type homeobox transcription factor 2 (CDX2) (+), thyroid transcription factor1 (TTF‐1) (+), Ki‐67 (70%), CD56 (−), P63 (−) and napsin‐A (−) (Figure 2C–F). Based on the pathological report, a primary HAL clinical stage IIb (T3N0M0) was diagnosed. Surgery was urgently indicated. To improve the prognosis, we formulated a combination regimen of albumin‐bound paclitaxel 300 mg + lobaplatin 50 mg + camrelizumab 200 mg combined with bevacizumab 500 mg on Day 1, once every 3 weeks. After four courses, the symptoms generally improved. CT scan results revealed that the size of the mass in the upper lobe of the left lung was smaller than before. Due to the patient's extensive formation of emphysema in the lungs, left upper lobectomy wedge resection was performed to completely extirpate the lesion by video‐assisted thoracic surgery. The postoperative pathological analysis revealed atypical hyperplasia of the partial alveolar epithelium; no tumour was clearly detected in the tissue or surgical margin. One month after the surgery, AFP was less than 0.75 IU/mL.

## 3 DISCUSSION

Hepatoid adenocarcinoma (HAC) is a rare primary extrahepatic organ or tissue with adenoid and the characteristics of hepatocellular differentiated adenocarcinoma, and its morphological and immunohistochemistry are similar to hepatocellular carcinoma (HCC). The most common site of HAC is the stomach, accounting for 63% of all cases, while HAL has an extremely low incidence, accounting for 5% of HAC cases.^7^

HAL is indicated mainly by imaging that shows inhomogeneous masses in the upper lung fields; enhanced CT scans reveal homogeneous or heterogeneous density.^5^, ^8^, ^9^ Thus, HAL is difficult to distinguish from other malignant tumours in the lung by imaging alone. Imaging studies can also detect lymph nodes or distant metastases, which aid in the evaluation of the clinical stage of the disease. Moreover, HAL is mainly diagnosed by histopathology and immunohistochemistry, which must rule out primary HCC or other HAC metastasis to the lungs.^9^, ^10^

Due to non‐specific clinical manifestations, HAL is often diagnosed at its advanced stage (stage III or IV), thereby resulting in shorter survival time.^7^ HAL patients who undergo surgery have an average life expectancy between 7 months and 7 years and a 1‐year survival rate of 55%.^11^

Early implementation of anatomic pneumonectomy is crucial for enhancing the survival rate of patients. Therefore, surgeons should actively participate in the assessment and functional evaluation of the patient's clinical stage and resectability, as well as determine surgical indications and techniques based on tumour progression and the patient's functional status.^12^ For patients with late‐stage HAL or unresectable tumour, chemoradiotherapy can be combined with other adjuvant therapy.^13^ Valle et al^14^ reported a case of stage IV HAL, with an overall survival of 55 months after four chemotherapy regimens, including cisplatin and pemetrexed with radiotherapy for metastatic lesions, highlighting the benefit of the combination of chemotherapy and radiotherapy. Basse et al^15^ reported a case of a partial response to immunotherapy despite a PD‐L1‐negative status, which subsequently resulted in death due to infectious complications. In the present case, neoadjuvant chemotherapy, immunotherapy and targeted therapy were started before surgery. After 3 months of treatment, imaging studies demonstrated efficacy. Subsequent postoperative pathology results confirmed complete remission.

To date, there is no preferred treatment prescription due to the challenges of organising large‐scale trials. More case reports are needed to provide guidance for the future treatment.

## AUTHOR CONTRIBUTIONS

Xuejian Sun was involved in drafting the manuscript. Jialin Liu and Ting Hu was involved in acquisition of data. Yefan Wu has gathered medical records. Hao Zhang designed and revised the manuscript. All authors have read and approved the final manuscript.

## CONFLICT OF INTEREST STATEMENT

The authors declare no conflict of interest in association with this report.

## ETHICS STATEMENT

The study was approved by the research ethics committee of the First Hospital of Lanzhou University (approval number: LDYYLL2023‐444) and did not need informed consent. Patient information was de‐identified for the purpose of this case report. Informed consent for publication was obtained from the patient.

## Data Availability

The data that support the findings of this study are available from the corresponding author upon reasonable request.
